# Interferon-β modulates microglial polarization to ameliorate delayed tPA-exacerbated brain injury in ischemic stroke

**DOI:** 10.3389/fimmu.2023.1148069

**Published:** 2023-03-31

**Authors:** Ping-Chang Kuo, Wen-Tsan Weng, Barbara A. Scofield, Hallel C. Paraiso, Paul Bojrab, Brandon Kimes, I-Chen Ivorine Yu, Jui-Hung Jimmy Yen

**Affiliations:** ^1^ Department of Microbiology and Immunology, Indiana University School of Medicine, Fort Wayne, IN, United States; ^2^ Department of Anatomy, Cell Biology and Physiology, Indiana University School of Medicine, Fort Wayne, IN, United States; ^3^ Doctor of Medicine Program, Indiana University School of Medicine, Fort Wayne, IN, United States

**Keywords:** tissue plasminogen activator, interferon beta, type I interferon receptor, blood brain barrier, microglial polarization, ischemic stroke

## Abstract

Tissue plasminogen activator (tPA) is the only FDA-approved drug for the treatment of ischemic stroke. Delayed tPA administration is associated with increased risks of blood-brain barrier (BBB) disruption and hemorrhagic transformation. Studies have shown that interferon beta (IFNβ) or type I IFN receptor (IFNAR1) signaling confers protection against ischemic stroke in preclinical models. In addition, we have previously demonstrated that IFNβ can be co-administered with tPA to alleviate delayed tPA-induced adverse effects in ischemic stroke. In this study, we investigated the time limit of IFNβ treatment on the extension of tPA therapeutic window and assessed the effect of IFNβ on modulating microglia (MG) phenotypes in ischemic stroke with delayed tPA treatment. Mice were subjected to 40 minutes transient middle cerebral artery occlusion (MCAO) followed by delayed tPA treatment in the presence or absence of IFNβ at 3h, 4.5h or 6h post-reperfusion. In addition, mice with MG-specific IFNAR1 knockdown were generated to validate the effects of IFNβ on modulating MG phenotypes, ameliorating brain injury, and lessening BBB disruption in delayed tPA-treated MCAO mice. Our results showed that IFNβ extended tPA therapeutic window to 4.5h post-reperfusion in MCAO mice, and that was accompanied with attenuated brain injury and lessened BBB disruption. Mechanistically, our findings revealed that IFNβ modulated MG polarization, leading to the suppression of inflammatory MG and the promotion of anti-inflammatory MG, in delayed tPA-treated MCAO mice. Notably, these effects were abolished in MG-specific IFNAR1 knockdown MCAO mice. Furthermore, the protective effect of IFNβ on the amelioration of delayed tPA-exacerbated ischemic brain injury was also abolished in these mice. Finally, we identified that IFNβ-mediated modulation of MG phenotypes played a role in maintaining BBB integrity, because the knockdown of IFNAR1 in MG partly reversed the protective effect of IFNβ on lessening BBB disruption in delayed tPA-treated MCAO mice. In summary, our study reveals a novel function of IFNβ in modulating MG phenotypes, and that may subsequently confer protection against delayed tPA-exacerbated brain injury in ischemic stroke.

## Introduction

Stroke is a leading cause of death and results in permanent disability in up to 30% of survivors. There are two types of strokes, ischemic stroke and hemorrhagic stroke, in which ischemic stroke accounts for more than 80% of stroke cases. Currently, tissue plasminogen activator (tPA) is the only FDA-approved drug for ischemic stroke. Mechanistically, tPA functions to dissolve blood clots that leads to reestablish the cerebral blood flow in the ischemic brain. However, tPA-induced reperfusion promotes the recruitment of peripheral inflammatory immune cells into the infarct core that subsequently results in the secondary brain injury (1). Furthermore, studies have shown that tPA induces microglia (MG) activation that exacerbates brain injury in ischemic stroke (2, 3). Moreover, the administration of tPA beyond its therapeutic window of 3-4.5h post-injury significantly increases the risk of blood brain barrier (BBB) disruption and hemorrhagic transformation (HT) after ischemic stroke (4–7).

MG activation plays an important role in the immunopathogenesis of the central nervous system (CNS) diseases. Following ischemic stroke, MG are rapidly activated and exert detrimental effects on ischemic brain injury. MG activation can be determined by detecting the expression of inflammatory markers, such as CD86 and CD16, and the upregulation of pro-inflammatory cytokines, including IL-1α, IL-β, TNF-α, and IL-6. Studies have shown that inflammatory MG markers and cytokines display an increased trend during the first 14 days following ischemic stroke (8). On the other hand, anti-inflammatory MG were also observed in the ischemic brain. Anti-inflammatory MG phenotype can be assessed by the expression of surface marker, macrophage mannose receptor 1 (CD206), and the upregulation of anti−inflammatory molecules, including IL− 10, arginase−1 (Arg1), Ym1, and TGF−β. Studies have shown that the expression of anti-inflammatory MG markers and molecules was increased at day 1 and peaked at day 5-7 post stroke. Importantly, anti-inflammatory MG exert protective effects on promoting brain repair and prognosis in ischemic stroke (8–10). Thus, modulating MG transformation from inflammatory to anti-inflammatory phenotype represents a critical strategy for attenuating neuroinflammation and ameliorating brain injury in ischemic stroke.

Interferon beta (IFNβ) is an FDA-approved therapy for the treatment of multiple sclerosis, and its immunomodulatory and anti-inflammatory properties were well characterized (11, 12). Hence, IFNβ exerts a potential to be utilized as an anti-inflammatory agent for the treatment of ischemic stroke. Indeed, we have previously demonstrated that IFNβ ameliorates brain injury through inhibiting MG activation and suppressing inflammatory immune cell infiltration of the CNS in stroke animals (13). In addition, studies from other groups showed that IFNβ and/or type I IFN receptor (IFNAR1) signaling confer protection against brain injury in ischemic stroke animal models (14–19). Moreover, our recent study showed that IFNβ can be co-administered with tPA to ameliorate delayed tPA-exacerbated brain injury in ischemic stroke (20). Collectively, studies from our and other groups demonstrate the beneficial effects of IFNβ treatment in ischemic stroke.

Our previous study demonstrated that IFNβ extended tPA therapeutic window to 3h post-injury in ischemic stroke in which IFNβ ameliorated delayed tPA-exacerbated ischemic brain injury and lessened tPA-aggravated BBB disruption and HT (20). In the present study, we investigated the time limit of IFNβ treatment on the extension of tPA therapeutic window and assessed the effect of IFNβ on the modulation of MG phenotypes in delayed tPA-treated stroke animals. Furthermore, mice with MG-specific IFNAR1 knockdown were generated to validate the effects of IFNβ on modulating MG phenotypes, ameliorating brain injury, and lessening BBB disruption in delayed tPA-treated stroke animals. In sum, our findings reveal that IFNβ exerts protective effects on modulating MG phenotypes, leading to the suppression of inflammatory MG and the induction of anti-inflammatory MG, and that subsequently confers protection against delayed tPA-exacerbated brain injury in ischemic stroke.

## Materials and methods

### Mice

Animal experimental procedures were approved by the Purdue Animal Care and Use Committee and performed in strict compliance with the National Institutes of Health Guide for the Care and Use of Laboratory Animals. C57BL/6, *Ifnar1^fl/fl^*, and *Cx3cr1^CreERT2/CreERT2^* mice were purchased from the Jackson Laboratory (Bar Harbor, ME) and bred in our animal facility. *Ifnar1^fl/fl^* mice were crossed with *Cx3cr1^CreERT2/CreERT2^* mice to generate *Ifnar1^fl/+^-Cx3cr1^CreERT2/+^* mice. *Ifnar1^fl/+^-Cx3cr1^CreERT2/+^* mice were then crossed with *Ifnar1^fl/+^-Cx3cr1^CreERT2/+^* mice to generate *Ifnar1^fl/fl^-Cx3cr1^CreERT2/+^* and *Cx3cr1^CreERT2/+^* mice. *Ifnar1^fl/fl^-Cx3cr1^CreERT2/+^* mice at 7-8 weeks old were subjected to *i.p*. injection of 75mg/kg Tamoxifen (TAM) for a total of 5 consecutive days to induce IFNAR1 knockdown in MG/macrophages. TAM-treated *Ifnar1^fl/fl^-Cx3cr1^CreERT2/+^* mice were housed for additional 7-8 weeks to allow peripheral monocyte/macrophages to be replenished and then subjected to ischemic stroke. *Cx3cr1^CreERT2/+^* control mice were subjected to the same procedure of TAM treatment followed by ischemic stroke induction. Mice were housed and bred with controlled humidity, temperature and 12h:12h light-dark cycle in the animal facility with food and water available ad libitum.

### Reagents

IFNβ was purchased from PBL Interferon Source (Piscataway, NJ), and tPA was purchased from Genentech (San Francisco, CA). Evans blue was purchased from Sigma-Aldrich (St. Louis, MO). Triphenyltetrazolium chloride (TTC) and trichloroacetic acid (TCA) were purchased from Alfa Aesar (Tewksbury, MA). Antibodies of Alexa Fluor 488 anti-CD45 (Clone: 30-F11), FITC anti-CD11a (Clone: M17/4), PE/Cy7 anti-Ly6C (Clone: HK1.4), APC anti-CD11b (Clone: M1/70), PE/Cy7 anti-CD68 (Clone: FA-11), PE/Cy7 anti-CD86 (Clone: GL-1), PE/Cy7 anti-CD206 (Clone: C068C2), APC anti-IFNAR1 (Clone: MAR1-5A3), and PE anti-IL-1α (Clone: ALF-161) were purchased from BioLegend (San Diego, CA). APC anti-Arg1 antibody was purchase from R&D Systems. APC anti-IL-1β (Clone: NJTEN3) antibody was purchase from eBioscience (Waltham, MA).

### Middle cerebral artery occlusion model

Cerebral ischemia was induced in 3-4 months old male and female mice as previously described (13, 20). Briefly, mice were subjected to intraluminal occlusion of right middle cerebral artery by the insertion of silicone-coated nylon monofilament (Doccol Corp, Sharon, MA). A laser doppler flowmetry was used to measure the cerebral blood flow (CBF). The filament was withdrawn to allow reperfusion after 40min or 4.5h occlusion. Animal’s body temperature was maintained at ~37°C throughout the surgery by a warming lamp and heating pad. Sham controls were subjected to the same surgical procedure without monofilament insertion. After surgery, mice were placed in the recovery cage in which the temperature was maintained at 37°C for 1h to recover from anesthesia. Mice that had a reduction of the CBF more than 80% during the occlusion were included in the study and assigned randomly to receive *i.v.* administration of vehicle (PBS), tPA (5mg/kg), or tPA+IFNβ (10,000U) at the indicated time points. tPA+IFNβ-treated MCAO mice received an additional dose of IFNβ at day 1 post-injury if they were sacrificed at day 2 or day 7. The doses of tPA and IFNβ used in this study were based on the previous studies (13, 20–22). The investigators who performed the experiments were blinded to the animal groups.

### Infarct volume measurements

Mice were perfused with PBS, and the ischemic brains were harvested and subjected to 2 mm coronal slicing with a rodent brain matrix. Brain sections were than stained with 1% TTC followed by scanning, and the infarct volumes were calculated by using ImageJ as previously described (13).

### Evans blue extravasation assay

Mice were *i.v.* administered 4 ml/kg 2% (w/v) Evans blue dye/0.9% saline solution through lateral tail vein. One hour after injection, mice were then anesthetized deeply and perfused with PBS to remove intravascular Evans blue. The brains were harvested and sliced. The brain sections were then subjected to scanning to obtain images. Subsequently, the hemispheres of brain sections were separated and weighted followed by homogenization with 50% TCA solution. After centrifugation, the supernatants were collected and diluted with 95% ethanol at the ratio of 1:3. The amount of extravascular Evans blue in the supernatants was then determined by measuring the fluorescence with excitation at 540/25nm and emission at 645/40nm using a BioTek Synergy HT microplate reader.

### Mononuclear cells isolation and FACS analysis

Mice subjected to MCAO were anesthetized deeply and transcardially perfused with PBS at indicated time points. The brains were harvested, and the meninge, olfactory bulb, and cerebellum were removed. The forebrains were then homogenized with 1x Hanks’ balanced salt solution (HBSS) buffer followed by filtration through a 70μm nylon cell strainer. Following centrifugation, cells were resuspended in 30% Percoll and underlayered with 70% Percoll. After centrifugation, the mononuclear cells were then isolated from the interface between 30% and 70% Percoll. For intracellular cytokine staining, the isolated cells were *ex-vivo* cultured in the presence of GolgiPlug for 4.5h. Cells were then collected and stained with antibodies of CD45 and CD11b followed by fixation and permeabilization. After wash, cells were stained with IL-1α and IL-1β antibodies followed by FACS analysis. For cell surface staining, the isolated cells were stained with antibodies of CD45 and CD11b in the presence of CD86 or CD11a and Ly6C antibodies followed by FACS analysis. For intracellular staining, the isolated cells were stained with antibodies of CD45 and CD11b followed by fixation and permeabilization. After wash, cells were stained with CD68, Arg1, or CD206 antibody followed by FACS analysis. The gating strategy of flow cytometry analysis to identify the population of MG with the intermediate expression of CD45 (CD45^int^) and positive expression of CD11b (CD11b^+^) is presented in **Supplementary Figure 1**. For *in vitro* intracellular cytokine staining, cells were treated with indicated conditions and harvested at the indicated time points. GolgiPlug was added to the culture 4.5h prior cell harvesting. Cells were then fixed and permeabilized, and subsequently subjected to IL-1β antibody staining followed by FACS analysis. For *in vitro* intracellular staining, cells treated with indicated conditions were harvested at the indicated time points followed by fixation and permeabilization. After wash, cells were then stained with Arg1 and CD206 antibodies followed by FACS analysis.

### Rotarod test

Motor coordination was assessed in MCAO mice by using the rotarod test (Model 47600, Ugo Basile, Varese, Italy). Prior to MCAO surgery, mice were trained on the rotarod apparatus with a setting of accelerating speed from 4 to 80 rpm over a 5min period for three trials with a 30min resting interval every day for three consecutive days. Following MCAO, mice were given three trials with a 30min resting interval at day 2, 4, and 6 post-injury. Motor function was assessed based on the latency to fall.

### Cell culture

Primary MG were generated from neonatal mice as previously described (23). Briefly, cerebral cortical cells were collected from 1-2 days old neonatal mice and seeded in T75 flasks containing Dulbecco’s modified eagle medium/nutrient mixture F-12 (DMEM/F12) complete media. After removing media, the flasks were replenished with complete media containing 10ng/ml GM-CSF on day 3 and 6 after plating. MG were harvested by shaking the flasks at 250rpm for 40min at 37°C on day 13 or 14. Cells were then seeded in cell culture wells followed by treatments.

### Quantitative polymerase chain reaction

mRNA expression was measured by Q-PCR analysis as previously described (24). The primers used were *Il-1α*: 5’-CGCTTGAGTCGGCAAAGAAAT-3’ and 5’-CTTCCCGTTGCTTGACGTT G-3’; *Il-1β*: 5’-CCCTGCAGCTGGAGAGTGTGGA-3’ and 5’-TGTGCTCTGCTTGTGAGGTG CTG-3’.

### Statistical analysis

All results in this study were given as mean ± SEM. Sample sizes were determined by power calculations based on our previous studies. The normal distribution of the data was confirmed by Shapiro-Wilk test. Comparisons among multiple groups were performed by one-way ANOVA (one variable) or two-way ANOVA (two variables) followed by Tukey *post hoc* test. Comparisons between two groups were performed by unpaired *t* test. Statistical analyses were performed by using GraphPad Prism 9 software. Statistical significance was determined as *p<0.05*.

## Results

### IFNβ confers protection against delayed tPA-exacerbated brain injury in ischemic stroke

**Figure 1:**
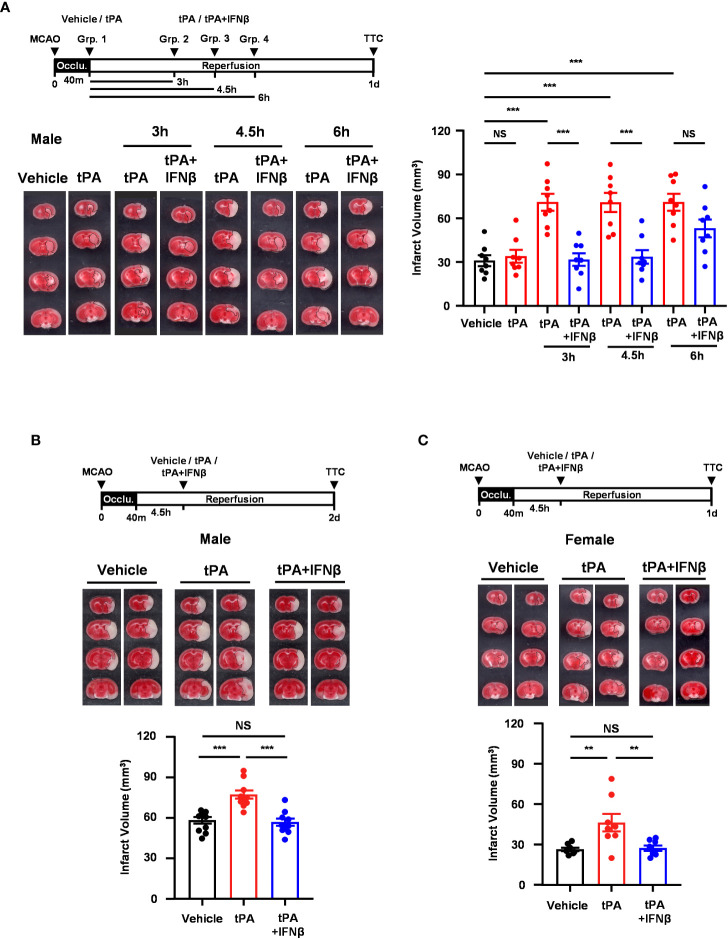
IFNβ confers protection against delayed tPA-exacerbated brain injury in ischemic stroke. **(A)** Male C57BL/6 mice were subjected to 40min MCAO followed by the administration of vehicle or tPA right after the onset of reperfusion or of tPA in the presence or absence of IFNβ at 3h, 4.5h, or 6h post-reperfusion (n=8/group). At day 1 post-injury, the ischemic brains were harvested and subjected to TTC staining. One representative TTC-stained brain sample of each group is shown, and the infarct volumes were also measured. **(B)** Male C57BL/6 mice subjected to 40min MCAO were administered vehicle, tPA, or tPA+IFNβ at 4.5h post-reperfusion (n=10/group). At day 2 post-injury, the ischemic brains were harvested and subjected to TTC staining. Two representative TTC-stained brain samples of each group are shown, and the infarct volumes were also measured. **(C)** Female C57BL/6 mice subjected to 40min MCAO were administered vehicle, tPA, or tPA+IFNβ at 4.5h post-reperfusion (n=8/group). At day 1 post-injury, the ischemic brains were harvested and subjected to TTC staining. Two representative TTC-stained brain samples of each group are shown, and the infarct volumes were measured. **p<0.01; ***p<0.001; NS, no significant difference by one-way ANOVA. Occlu., occlusion; Grp., group.

We have previously demonstrated that delayed but not early tPA treatment exacerbated brain injury in ischemic stroke, and IFNβ ameliorated ischemic brain injury in MCAO mice subjected to delayed tPA treatment (20). In this study, we aimed to investigate the time limit of IFNβ on the extension of tPA therapeutic window in ischemic stroke. As we have previously shown that IFNβ administered at 3h post-reperfusion ameliorated ischemic brain injury (13), we first assessed the time limit of IFNβ-conferred protection against ischemic brain injury. C57BL/6 mice were subjected to 40min MCAO followed by IFNβ administration at 4.5h or 6h post-reperfusion, and MCAO mice were sacrificed at day 2 post-injury to determine the level of brain injury. Our results showed that IFNβ administered at 4.5h but not 6h post-reperfusion significantly attenuated infarct volumes in MCAO mice (**Supplementary Figure 2**). We then determined the therapeutic potential of IFNβ on the extension of tPA therapeutic window in MCAO mice at different time points. Mice subjected to 40min MCAO were treated with tPA at 40min post-injury or with tPA in the presence or absence of IFNβ at 3h, 4.5h, or 6h post-reperfusion. Consistent with our previous findings (20), tPA administered at 40min post-injury did not alter the level of brain injury in MCAO mice compared to vehicle-treated MCAO controls. However, delayed tPA treatment at 3h, 4.5h, or 6h post-reperfusion exacerbated brain injury in MCAO mice, and the level of brain injury was comparable among these animals at day 1 post-injury (**Figure 1A**). Importantly, we found that IFNβ attenuated ischemic brain injury-exacerbated by delayed tPA treatment at 3h and 4.5h post-reperfusion (**Figure 1A**). Although there was a trend that IFNβ reduced brain infarct in MCAO mice treated with tPA at 6h post-reperfusion, it did not reach statistical significance (**Figure 1A**). Furthermore, the detrimental effect of delayed tPA treatment at 4.5h post-reperfusion on ischemic brain injury and the protective effect of IFNβ on delayed tPA-exacerbated brain injury were observed in MCAO mice at day 2 post-injury (**Figure 1B**). Finally, we investigated the observed effects in female MCAO mice. Our results showed that delayed tPA treatment at 4.5h post-reperfusion exacerbated brain injury, and consistently IFNβ ameliorated delayed tPA-exacerbated brain injury in female MCAO mice (**Figure 1C**). Notably, we observed a smaller infarct in vehicle-treated female MCAO mice compared to vehicle-treated male MCAO mice. Similarly, delayed tPA-exacerbated brain injury was less severe in female MCAO mice compared to male MCAO mice (**Figures 1A** vs. **1C**). These results suggest that female mice exhibit less severe brain injury compared to male mice following ischemic stroke, consistent with the previous findings (25, 26). Collectively, our results demonstrate that delayed tPA treatment exacerbates ischemic brain injury, and IFNβ exerts a therapeutic potential to extend tPA therapeutic window to 4.5h post-injury and ameliorate delayed tPA-exacerbated brain injury in ischemic stroke.

### IFNβ confers long-term protection against ischemic stroke with delayed tPA treatment

**Figure 2:**
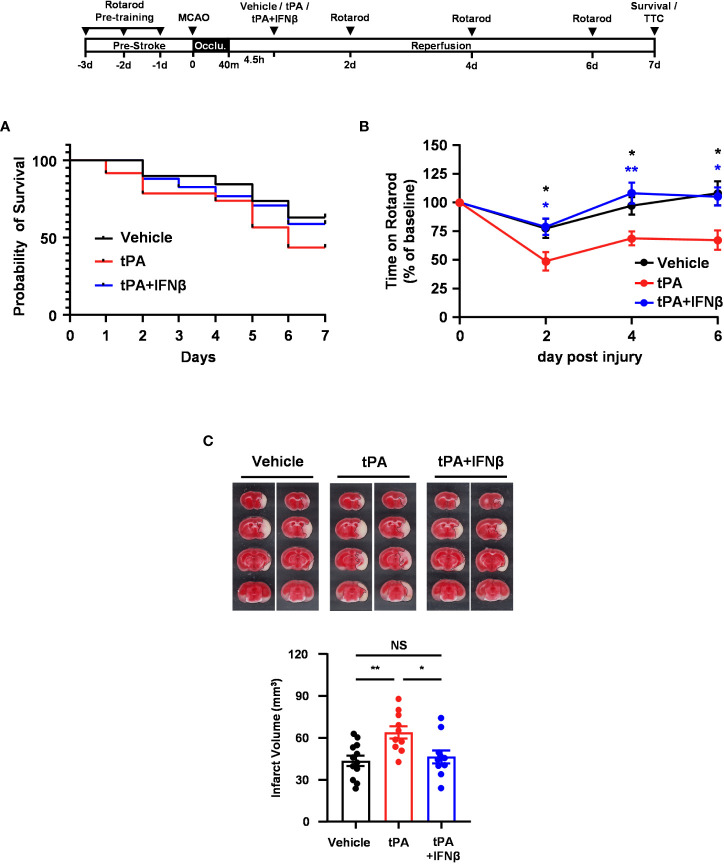
IFNβ confers long-term protection against ischemic stroke with delayed tPA treatment. Male C57BL/6 mice were subjected to 40min MCAO followed by the administration of vehicle, tPA, or tPA+IFNβ at 4.5h post-reperfusion. MCAO mice were then subjected to **(A)** survival assay for 7 days and **(B)** rotarod tests at day 2, 4, and 6 post-injury. *p<0.05, **p<0.01 compared to tPA by unpaired *t*-test. **(C)** At day 7 post-injury, the survived MCAO mice (vehicle: n=12; tPA: n=10; tPA+IFNβ: n=10) were sacrificed and subjected to TTC staining. Two representative TTC-stained samples of each group are shown, and the infarct volumes were measured. *p<0.05; **p<0.01; NS, no significant difference by one-way ANOVA.

To further evaluate whether IFNβ conferred long-term protection against ischemic stroke with delayed tPA treatment, mice were subjected to 40min MCAO followed by vehicle, tPA, or tPA+IFNβ administration at 4.5h post-reperfusion, and the survival assay was performed. Our results showed that tPA-treated MCAO mice displayed reduced survival compared to vehicle-treated MCAO mice at day 7 post-injury. Notably, tPA+IFNβ-treated MCAO mice exhibited increased survival compared to tPA-treated MCAO mice and displayed a similar level of survival as vehicle-treated MCAO mice at day 7 post-injury (**Figure 2A**). Furthermore, the rotarod test was performed to assess the effect of delayed tPA treatment in the presence or absence of IFNβ on motor coordination in MCAO mice. Our results showed that tPA-treated MCAO mice had a poor performance on the rotarod test compared to vehicle-treated MCAO mice. In contrast, tPA+IFNβ-treated MCAO mice exhibited an improved performance on the rotarod test compared to tPA-treated MCAO mice (**Figure 2B**). Finally, we assessed the level of brain injury in MCAO mice treated with delayed tPA in the presence or absence of IFNβ at day 7 post-injury. Our results showed that tPA+IFNβ-treated MCAO mice exhibited reduced infarct volumes compared to tPA-treated MCAO mice, and the infarct volumes were comparable between vehicle- and tPA+IFNβ-treated MCAO mice (**Figure 2C**). Altogether, our results demonstrate that IFNβ confers long-term protection against ischemic stroke with delayed tPA treatment, and that was accompanied with attenuated brain infarct volumes, improved motor coordination, and increased survival.

### IFNβ modulates MG polarization in delayed tPA-treated ischemic stroke

**Figure 3:**
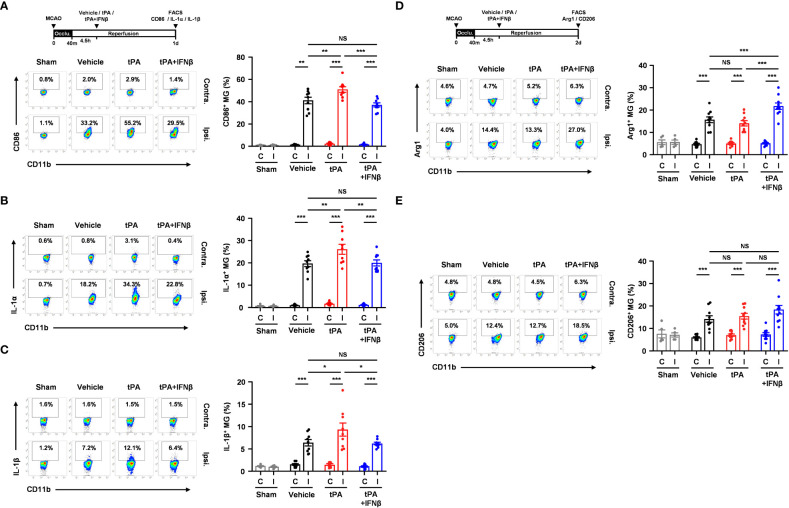
IFNβ modulates MG polarization in delayed tPA-treated ischemic stroke. Male C57BL/6 mice were subjected to sham or 40min MCAO followed by the administration of vehicle, tPA, or tPA+IFNβ at 4.5h post-reperfusion. **(A–C)** At day 1 post-injury, the contralateral (Contra.; C) and ipsilateral (Ipsi.; I) hemispheres of sham (n=6), and vehicle-, tPA-, and tPA+IFNβ-treated MCAO mice (n=9/group) were harvested followed by mononuclear cell isolation. **(A)** The isolated cells were stained with CD45 and CD11b antibodies in the presence of CD86 antibody to determine the surface expression of CD86 in CD45^int^CD11b^+^ MG. **(B, C)** The isolated mononuclear cells were ex-vivo cultured in the presence of GolgiPlug for 4.5h followed by staining with CD45 and CD11b antibodies. After fixation and permeabilization, cells were then stained with IL-1α and IL-1β antibodies to determine the intracellular expression of IL-1α and IL-1β in CD45^int^CD11b^+^ MG by flow cytometry. **(D, E)** At day 2 post-injury, the isolated mononuclear cells were stained with CD45 and CD11b antibodies. Following fixation and permeabilization, cells were then stained with Arg1 and CD206 antibodies to determine the expression of Arg1 and CD206 in CD45^int^CD11b^+^ MG by flow cytometry. *p<0.05; **p<0.01; ***p<0.001; NS, no significant difference by two-way ANOVA.

To elucidate whether IFNβ modulated MG phenotypes to confer protection against delayed tPA-exacerbated ischemic brain injury, we assessed MG inflammatory and anti-inflammatory phenotypes in 40min MCAO mice treated with vehicle, tPA, or tPA+IFNβ at 4.5h post-reperfusion. MG were determined as CD45^int^CD11b^+^ cells in the ischemic brain. As previous studies showed that CD45^high^ (CD45^hi^) infiltrating monocytes/macrophages might become CD45^int^ MG-like phenotypes following ischemic brain injury (27–29), we measured the expression of macrophage markers, CD11a and Ly6C, in the population of CD45^int^CD11b^+^ cells in the ischemic brain. We found that CD45^int^CD11b^+^ cells did not express CD11a and Ly6C in the ischemic brain of MCAO mice at day 1 post-injury (**Supplementary Figure 3A**). We then determined MG phenotypes in the ischemic brain of vehicle-, tPA-, and tPA+IFNβ-treated MCAO mice. Our results showed that ischemic stroke induced MG activation, and that was accompanied with increased CD86 expression and upregulated IL-1α and IL-1β production in the ipsilateral hemisphere of vehicle-treated MCAO mice compared to the ipsilateral hemisphere of sham controls and the contralateral hemisphere of vehicle-treated MCAO mice at day 1 post-injury (**Figures 3A–C**). Notably, delayed tPA treatment aggravated MG inflammatory phenotype, because the expression of CD86, IL-1α, and IL-1β in MG was further upregulated in the ipsilateral hemisphere of tPA-treated MCAO mice compared to that of vehicle-treated MCAO mice (**Figures 3A–C**). Importantly, IFNβ was able to suppress delayed tPA-induced CD86, IL-1α, and IL-1β upregulation in MG following MCAO (**Figures 3A–C**).

We then evaluated whether IFNβ promoted MG anti-inflammatory phenotype to confer protection against delayed tPA-exacerbated ischemic brain injury. The expression of anti-inflammatory molecules, Arg1 and CD206, and anti-inflammatory cytokine IL-10 in MG was assessed in MCAO mice treated with vehicle, tPA, or tPA+IFNβ at 4.5h post-reperfusion. We found ischemic stroke enhanced MG Arg1 and CD206 expression in the ipsilateral hemisphere of vehicle-treated MCAO mice compared to that of sham controls at day 2 post-injury (**Figures 3D, E**). However, delayed tPA treatment did not alter MG Arg1 and CD206 expression compared to vehicle treatment in MCAO mice. Importantly, IFNβ upregulated MG Arg1 expression, as the expression level of Arg1 in MG was higher in the ipsilateral hemisphere of tPA+IFNβ-treated MCAO mice than that of tPA-treated MCAO mice (**Figure 3D**). Interestingly, the frequency of Ly6C^+^ cells in the CD45^int^CD11b^+^ population was higher in tPA-treated MCAO mice than vehicle- or tPA+IFNβ-treated MCAO mice, although it did not reach statistically significant differences (**Supplementary Figure 3B**). However, we found Arg1^+^ cells was mostly associated with Ly6C^-^CD45^int^CD11b^+^ cells but not Ly6C^+^CD45^int^CD11b^+^ cells in the ischemic brain of tPA+IFNβ-treated MCAO mice (**Supplementary Figure 3C**), suggesting IFNβ mainly modulates the phenotype of resident MG in the ischemic brain. Finally, we observed a trend that IFNβ enhanced MG CD206 expression in tPA+IFNβ-treated MCAO mice compared to tPA-treated MCAO mice (**Figure 3E**). However, we did not observe an induction of IL-10 in MG in vehicle, tPA, or tPA+IFNβ-treated MCAO mice (Data not shown). Collectively, our results demonstrate that delayed tPA treatment aggravates MG inflammatory phenotype; in contrast, IFNβ diminishes delayed tPA-aggravated MG inflammatory phenotype in ischemic stroke. Furthermore, we identify that IFNβ promotes MG anti-inflammatory phenotype in delayed tPA-treated ischemic stroke. Thus, our results suggest that IFNβ exerts protective effects in ischemic stroke with delayed tPA treatment through suppressing tPA-aggravated inflammatory MG and promoting anti-inflammatory MG in the ischemic brain.

### IFNβ-conferred protection against delayed tPA-exacerbated ischemic brain injury is abolished in MCAO mice with MG-specific IFNAR1 knockdown

**Figure 4:**
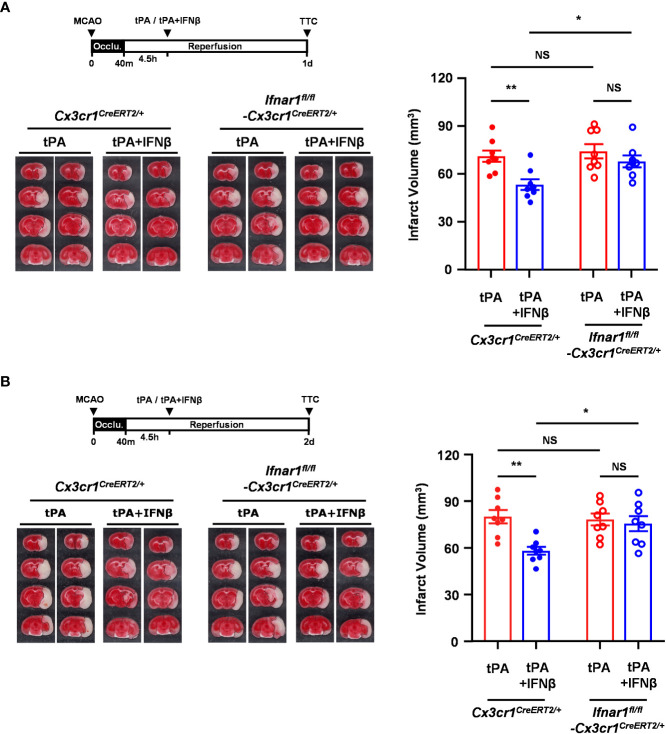
IFNβ-conferred protection against delayed tPA-exacerbated ischemic brain injury is abolished in MCAO mice with MG-specific IFNAR1 knockdown. Male *Cx3cr1^CreERT2/+^* and *Ifnar1^fl/fl^-Cx3cr1^CreERT2/+^* mice were subjected to 40min MCAO followed by tPA or tPA+IFNβ treatment at 4.5h post-reperfusion (n=8/group). **(A)** At day 1 or **(B)** day 2 post-injury, the ischemic brains were harvested and subjected to TTC staining. Two representative TTC-stained brain samples of each group are shown, and the infarct volumes were also measured. *p<0.05; **p<0.01; NS, no significant difference by two-way ANOVA.

Since we observed IFNβ modulated MG phenotypes and ameliorated brain injury in delayed tPA-treated MCAO mice, we thought to assess whether IFNβ-mediated activation of IFNAR1 signaling in MG plays an essential role in ameliorating delayed tPA-exacerbated brain injury in ischemic stroke. Thus, we generated *Ifnar1^fl/fl^-Cx3cr1^CreERT2/+^* mice and subjected mice to TAM treatment to induce IFNAR1 knockdown specifically in MG (**Supplementary Figure 4A**). *Ifnar1^fl/fl^-Cx3cr1^CreERT2/+^* and control *Cx3cr1^CreERT2/+^* mice were then subjected to 40min MCAO followed by tPA or tPA+IFNβ treatment at 4.5h post-reperfusion, and sacrificed at day 1 or day 2 post-injury to assess the level of brain injury. We confirmed whether IFNAR1 was deleted from MG in MCAO mice. Our results showed that MG in the ischemic brain of tPA- and tPA+IFNβ-treated control *Cx3cr1^CreERT2/+^* MCAO mice expressed a comparable level of IFNAR1. As expected, MG in the ischemic brain of tPA- and tPA+IFNβ-treated *Ifnar1^fl/fl^-Cx3cr1^CreERT2/+^* MCAO mice exhibited IFNAR1 knockdown (**Supplementary Figure 4B**). We then compared the level of brain injury in *Cx3cr1^CreERT2/+^* and *Ifnar1^fl/fl^-Cx3cr1^CreERT2/+^* MCAO mice subjected to tPA or tPA+IFNβ treatment. Our results showed that IFNβ ameliorated delayed tPA-exacerbated brain injury in *Cx3cr1^CreERT2/+^* MCAO mice at day 1 and day 2 post-injury (**Figures 4A, B**), and these findings are consistent with the results of C57BL/6 MCAO mice treated with delayed tPA in the presence or absence of IFNβ (**Figure 1B**). In contrast, the protective effect of IFNβ on ameliorating delayed tPA-exacerbated brain injury was abolished in *Ifnar1^fl/fl^-Cx3cr1^CreERT2/+^* MCAO mice (**Figures 4A, B**). Collectively, our results demonstrate that IFNβ-induced IFNAR1 signaling activation in MG plays an essential role in conferring protection against delayed tPA-exacerbated brain injury in ischemic stroke.

### MG-specific IFNAR1 knockdown abolishes IFNβ-mediated modulation of MG phenotypes in delayed tPA-treated ischemic stroke

**Figure 5:**
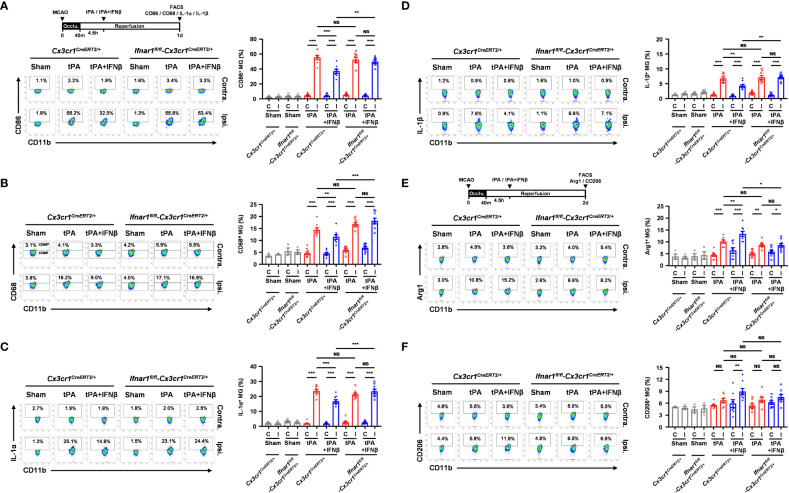
MG-specific IFNAR1 knockdown abolishes IFNβ-mediated modulation of MG phenotypes in delayed tPA-treated ischemic stroke. Male *Cx3cr1^CreERT2/+^* and *Ifnar1^fl/fl^-Cx3cr1^CreERT2/+^* mice were subjected to sham (n=3-4) or 40min MCAO followed by *i.v.* administration of tPA or tPA+IFNβ at 4.5h post-reperfusion (n=8/group). **(A, B)** At day 1 post-injury, the contralateral (Contra.; C) and ipsilateral (Ipsi.; I) hemispheres of sham, and tPA- and tPA+IFNβ-treated MCAO mice were harvested followed by mononuclear cell isolation. The isolated mononuclear cells were stained with CD45 and CD11b antibodies followed by surface staining of CD86 antibody or intracellular staining of CD68 antibody. The expression of CD86 and CD68 in CD45^int^CD11b^+^ MG was then determined by flow cytometry. The gating of CD68 low (CD68^L^) was based on the basal expression of CD68 in MG in sham controls, and the expression level of CD68 higher than CD68^L^ was then determined as CD68 high (CD68^H^). **(C, D)** The isolated cells were *ex-vivo* cultured in the presence of GolgiPlug for 4.5h and then stained with CD45 and CD11b antibodies. Following fixation and permeabilization, cells were subjected to the staining of IL-1α and IL-1β antibodies to determine the intracellular expression of IL-1α and IL-1β in CD45^int^CD11b^+^ MG by flow cytometry. **(E, F)** At day 2 post-injury, the isolated mononuclear cells were stained with CD45 and CD11b antibodies followed by fixation and permeabilization. The isolated cells were then stained with Arg1 and CD206 antibodies followed by flow cytometry analysis to assess the expression of Arg1 and CD206 in CD45^int^CD11b^+^ MG. *p<0.05; **p<0.01; ***p<0.001; NS, no significant difference by two-way ANOVA.

To determine whether IFNβ failed to provide a protective effect in MG-specific IFNAR1 knockdown MCAO mice with delayed tPA treatment was due to the abrogated effect of IFNβ on modulating MG phenotypes, *Ifnar1^fl/fl^-Cx3cr1^CreERT2/+^* and *Cx3cr1^CreERT2/+^* mice were subjected to 40min MCAO followed by tPA or tPA+IFNβ treatment at 4.5h post-reperfusion and the ischemic brains were then harvested to assess the expression of inflammatory and anti-inflammatory molecules in MG. Our results showed that the inflammatory molecules, CD86 and CD68, and the inflammatory cytokines, IL-1α and IL-1β, were suppressed in MG in the ipsilateral hemisphere of tPA+IFNβ-treated *Cx3cr1^CreERT2/+^* MCAO mice compare to that of tPA-treated *Cx3cr1^CreERT2/+^* MCAO mice (**Figures 5A–D**). These results are consistent with the results of C57BL/6 MCAO mice subjected to delayed tPA treatment in the presence or absence of IFNβ (**Figures 3A–C**). On the contrary, IFNβ-mediated suppression of delayed tPA-enhanced CD86, CD68, IL-1α, and IL-1β expression in MG was abolished in *Ifnar1^fl/fl^-Cx3cr1^CreERT2/+^* MCAO mice (**Figures 5A–D**). Furthermore, we observed that the expression of anti-inflammatory marker Arg1 in MG was upregulated in tPA+IFNβ-treated *Cx3cr1^CreERT2/+^* MCAO mice compared to tPA-treated *Cx3cr1^CreERT2/+^* MCAO mice (**Figure 5E**), consistent with the results observed in C57BL/6 MCAO mice (**Figure 3D**). Similar to C57BL/6 MCAO mice, there was a trend of CD206 upregulation in tPA+IFNβ-treated *Cx3cr1^CreERT2/+^* MCAO mice compared to tPA-treated *Cx3cr1^CreERT2/+^* MCAO mice (**Figure 5F**). However, IFNβ-mediated upregulation of Arg1 and CD206 expression in MG was abolished in *Ifnar1^fl/fl^-Cx3cr1^CreERT2/+^* MCAO mice with tPA treatment (**Figures 5E, F**). Altogether, our results demonstrate that the knockdown of IFNAR1 specifically in MG abolishes IFNβ-mediated modulation of MG phenotypes that may subsequently abrogate the protective effect of IFNβ on the amelioration of delayed tPA-exacerbated brain injury in ischemic stroke.

### IFNβ inhibits inflammatory MG and promotes anti-inflammatory MG *in vitro*

**Figure 6:**
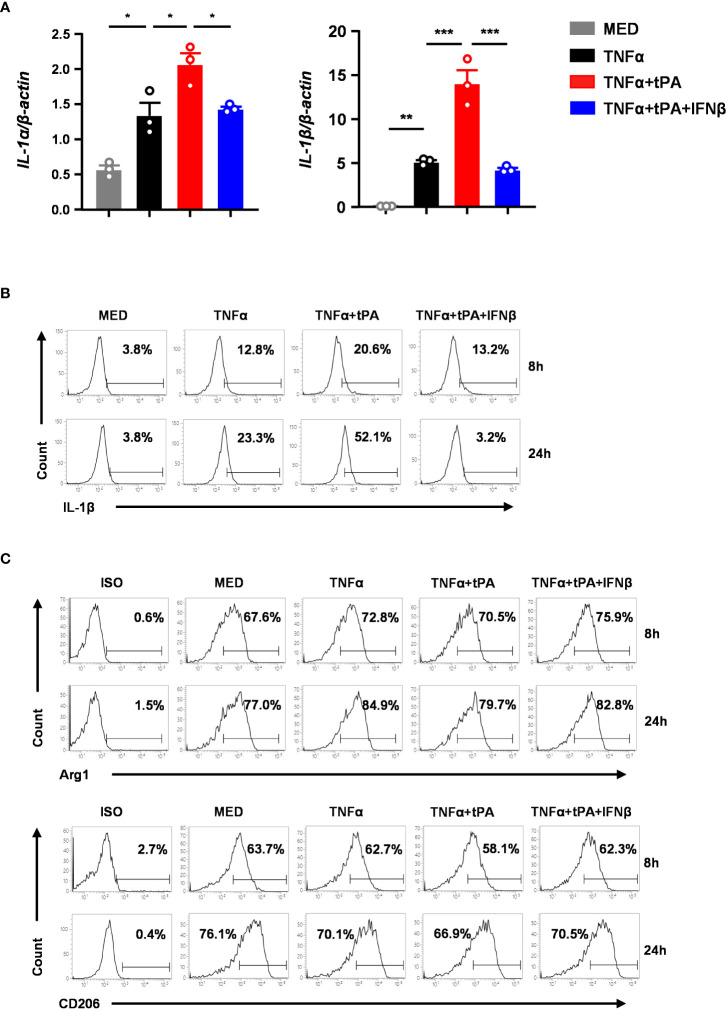
IFNβ inhibits inflammatory MG and promotes anti-inflammatory MG *in vitro.* Primary MG were treated with TNFα (50ng/ml) or TNFα+tPA (10µg/ml) in the presence or absence of IFNβ (1000U/ml). **(A)** At 8h after treatment, cells were harvested and subjected to Q-PCR analysis for IL-1α and IL-1β mRNA expression (n=3 technique replicates per group). The results represent 3-4 independent biological replicates with similar results. *p<0.05; **p<0.01; ***p<0.001 by one-way ANOVA. **(B)** At 8h and 24h post-treatment, cells were fixed and permeabilized followed by intracellular staining of IL-1β. The frequency (%) of IL-1β expression was measured. The experiments were repeated for 6 times with similar results obtained. **(C)** At 8h and 24h post-treatment, cells were fixed and permeabilized followed by staining of Arg1 and CD206. The frequency (%) of Arg1 and CD206 expression was measured. The experiments were repeated for 3 times with similar results obtained.

To confirm our *in vivo* findings that IFNβ modulated MG phenotypes in tPA-treated MCAO mice, we assessed the effect of tPA and IFNβ on regulating the expression of inflammatory and anti-inflammatory molecules in primary MG. Because studies reported that TNFα was induced in the ischemic brain to induce neuroinflammation (20, 30–32), we therefore activated MG with TNFα or TNFα+tPA in the presence or absence of IFNβ followed by Q-PCR and flow cytometry analysis to assess the expression of inflammatory and ant-inflammatory molecules. Our results showed that TNFα upregulated IL-1α and IL-1β mRNA expression in MG, and tPA further enhanced the expression of these inflammatory cytokines, indicating tPA promotes MG activation (**Figure 6A**). Importantly, IFNβ attenuated IL-1α and IL-1β mRNA expression in TNFα+tPA-treated MG (**Figure 6A**). Our flow cytometry analysis confirmed that tPA enhanced IL-1β expression in TNFα-treated MG, and IFNβ suppressed TNFα+tPA-induced IL-1β expression in MG (**Figure 6B**). Furthermore, we assessed the effect of IFNβ on the expression of anti-inflammatory molecules in MG. Our results showed that IFNβ upregulated Arg1 and CD206 expression in TNFα+tPA-treated MG (**Figure 6C**). Collectively, our results demonstrate that IFNβ suppresses inflammatory cytokine expression and promotes anti-inflammatory molecule expression in TNFα+tPA-treated MG *in vitro*, and that confirms our *in vivo* observation of IFNβ-mediated suppression of inflammatory MG and promotion of anti-inflammatory MG in tPA-treated MCAO mice.

### IFNβ confers protection against delayed tPA-exacerbated brain injury and BBB disruption in ischemic stroke subjected to 4.5h occlusion

**Figure 7:**
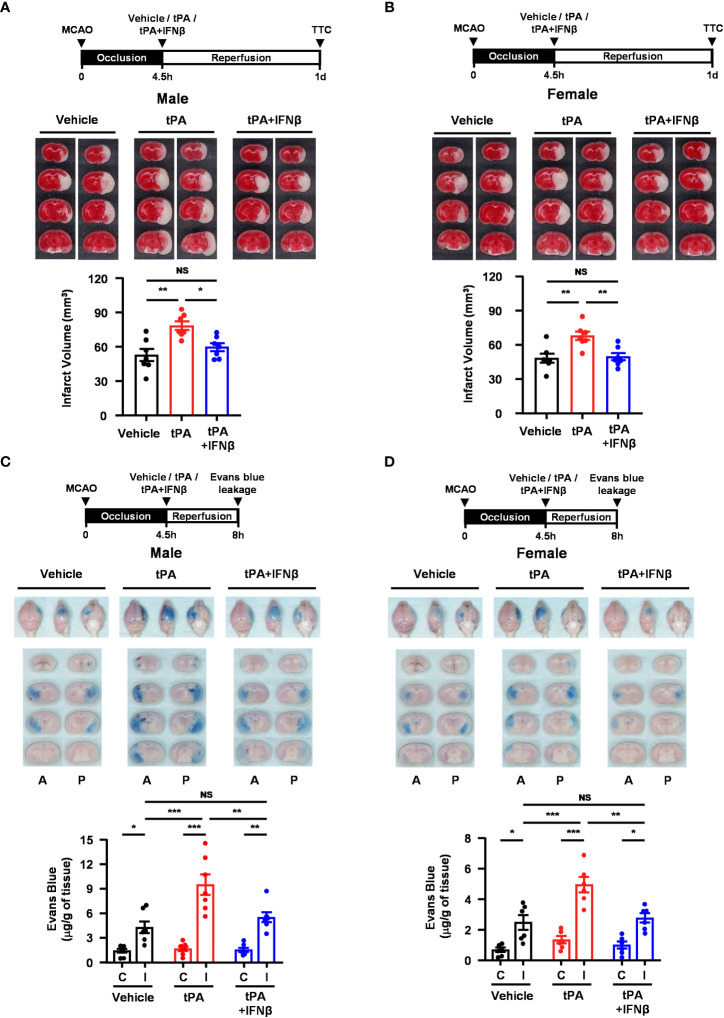
IFNβ confers protection against delayed tPA-exacerbated brain injury and BBB disruption in ischemic stroke subjected to 4.5h occlusion. **(A)** Male and **(B)** female C57BL/6 mice were subjected to 4.5h MCAO followed immediately by vehicle, tPA, or tPA+IFNβ administration. At day 1 post-injury, the ischemic brains were harvested and subjected to TTC staining. Two representative TTC-stained brain samples of each group are shown, and the infarct volumes were measured (n=7/group). *p<0.05; **p<0.01; NS: no significant difference by one-way ANOVA. **(C)** Male and **(D)** female C57BL/6 mice were subjected to 4.5h MCAO followed immediately by *i.v.* administration of vehicle, tPA, or tPA+IFNβ. Mice were injected with Evans blue 1h prior to euthanasia, and the ischemic brains were harvested at 8h post-injury followed by imaging and sectioning. The Evans blue leakage in the contralateral and ipsilateral hemispheres was quantified (male: n=7/group; female: n=6/group). A, anterior surface; P, posterior surface; C, contralateral hemisphere; I, ipsilateral hemisphere. *p<0.05; **p<0.01; ***p<0.001; NS, no significant difference by two-way ANOVA.

We observed that IFNβ conferred protection against tPA-exacerbated brain injury in 40min MCAO mice with delayed tPA treatment at 4.5h post-reperfusion. To closely mimic the clinical condition, we subjected mice to a long-term occlusion of 4.5h followed immediately by vehicle, tPA, or tPA+IFNβ treatment. The ischemic brains were harvested at day 1 post-injury to assess the severity of brain injury. Our results showed that tPA treatment at 4.5h post-injury exacerbated brain injury in MCAO mice, and importantly IFNβ was able to ameliorate delayed tPA-exacerbated ischemic brain injury (**Figure 7A**). Similarly, we observed that tPA treatment at 4.5h post-injury exacerbated brain injury in female 4.5h MCAO mice, and IFNβ attenuated delayed tPA-exacerbated brain injury in female MCAO mice (**Figure 7B**). In addition, tPA administration beyond its therapeutic window has been shown to aggravate BBB disruption in ischemic stroke (20). We therefore assessed whether delayed tPA treatment at 4.5h post-injury aggravated BBB disruption and whether IFNβ lessened delayed tPA-aggravated BBB disruption in 4.5h MCAO mice. Indeed, our results showed that delayed tPA treatment aggravated BBB disruption, leading to enhanced Evans blue leakage in the ipsilateral hemisphere of tPA-treated MCAO mice compared to that of vehicle-treated MCAO mice (**Figure 7C**). Importantly, we observed IFNβ lessened delayed tPA-aggravated BBB disruption, resulting in attenuated Evans blue leakage in the ipsilateral hemisphere of tPA+IFNβ-treated MCAO mice compared to that of tPA-treated MCAO mice (**Figure 7C**). Furthermore, we assessed whether IFNβ offered protection against delayed tPA-aggravated BBB disruption in female MCAO mice. Although the level of BBB disruption was less severe in female MCAO mice compared to male MCAO mice because of sex-mediated differences in ischemic brain injury (25, 26), our results showed that delayed tPA treatment aggravated BBB disruption and IFNβ lessened delayed tPA-aggravated BBB disruption in female MCAO mice (**Figure 7D**), consistent with the results of male MCAO mice (**Figure 7C**). Altogether, our results demonstrate that IFNβ ameliorates delayed tPA-exacerbated brain injury and lessens delayed tPA-aggravated BBB disruption in MCAO mice subjected to 4.5h occlusion.

### MG-specific IFNAR1 knockdown partly reverses IFNβ-lessened delayed tPA-aggravated BBB disruption in ischemic stroke

**Figure 8:**
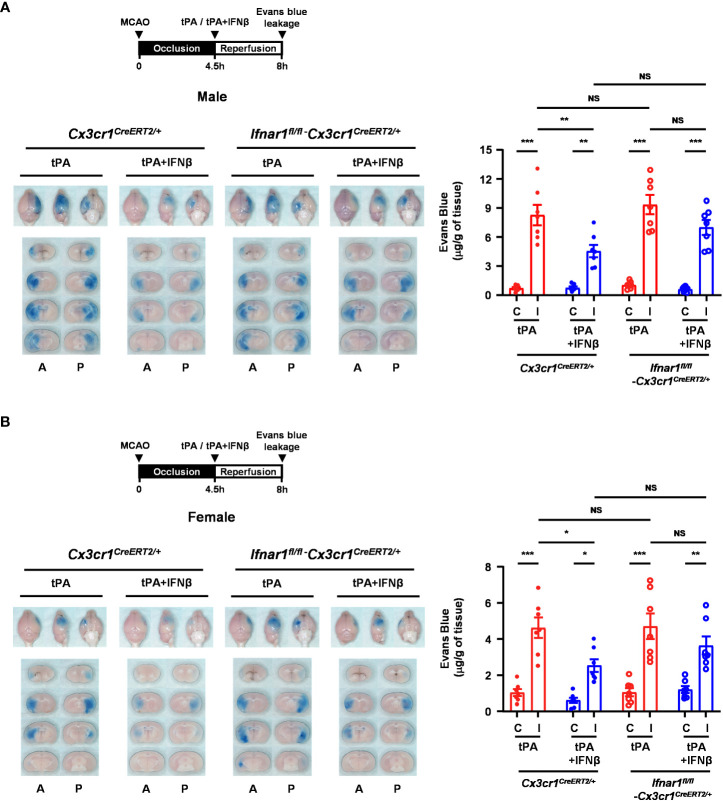
MG-specific IFNAR1 knockdown partly reverses IFNβ-lessened delayed tPA-aggravated BBB disruption in ischemic stroke. **(A)** Male and **(B)** female *Cx3cr1^CreERT2/+^* and *Ifnar1^fl/fl^-Cx3cr1^CreERT2/+^* mice were subjected to 4.5h MCAO followed immediately by tPA or tPA+IFNβ administration. Mice were then injected with Evans blue 1h prior to euthanasia, and the ischemic brains were harvested at 8h post-injury followed by imaging and sectioning. The Evans blue leakage in the contralateral and ipsilateral hemispheres was quantified (male: n=7/group; female: n=7/group). A, anterior surface; P, posterior surface; C, contralateral hemisphere; I, ipsilateral hemisphere. *p<0.05; **p<0.01; ***p<0.001; NS, no significant difference by two-way ANOVA.

To further investigate whether IFNβ-mediated modulation of MG phenotypes contributes to the protective effect of IFNβ on lessening delayed tPA-aggravated BBB disruption in ischemic stroke, we assessed the severity of BBB disruption in *Ifnar1^fl/fl^-Cx3cr1^CreERT2/+^* and *Cx3cr1^CreERT2/+^* MCAO mice treated with delayed tPA in the presence or absence of IFNβ. Our results showed that IFNβ lessened BBB disruption in tPA-treated *Cx3cr1^CreERT2/+^* MCAO mice, resulting in attenuated Evans blue leakage in the ischemic brain of tPA+IFNβ-treated *Cx3cr1^CreERT2/+^* MCAO mice compared to that of tPA-treated *Cx3cr1^CreERT2/+^* MCAO mice (**Figure 8A**). Interestingly, we found that the protective effect of IFNβ on lessening delayed tPA-aggravated BBB disruption was partly but not fully reversed in *Ifnar1^fl/fl^-Cx3cr1^CreERT2/+^* MCAO mice, as there was no statistically significant difference in the Evans blue leakage between *Ifnar1^fl/fl^-Cx3cr1^CreERT2/+^* and *Cx3cr1^CreERT2/+^* MCAO mice treated with tPA+IFNβ (**Figure 8A**). The similar results were also observed in female MCAO mice (**Figure 8B**). Collectively, our results suggest that IFNβ-mediated modulation of MG phenotypes may partly contribute to lessening delayed tPA-aggravated BBB disruption in ischemic stroke.

## Discussion

The use of tPA is limited by its narrow therapeutic window of 3 to 4.5h post-onset of ischemic stroke, and the administration of tPA beyond its therapeutic window has been linked to increased risks of aggravated BBB disruption and HT induction. Studies have reported that only less than 10% of patients with acute ischemic stroke were able to receive tPA treatment (33, 34). Thus, developing a therapy that can be co-administered with tPA to ameliorate ischemia-induced neuroinflammation and extend tPA therapeutic window would be of clinical importance. We have previously demonstrated that IFNβ conferred protection against ischemic stroke (13). In our follow-up study, we further demonstrated that IFNβ was able to extend tPA therapeutic window to 3h post-injury. Mechanistically, we identified that IFNβ attenuated delayed tPA-induced brain injury exacerbation, BBB disruption aggravation, and HT induction in ischemic stroke, and these protective effects were partly mediated through IFNβ-exerted suppression of MMP3 and MMP9 in the ischemic brain (20). In the present study, we further investigated the time limit of IFNβ on the extension of tPA therapeutic window in ischemic stroke. Our results demonstrated that IFNβ was able to extend tPA therapeutic window to 4.5h post-injury and that was accompanied with ameliorated brain injury and lessened BBB disruption in ischemic stroke. These findings are clinically significant. As previous studies reported that the rodent time is about 40 times faster than human time and one human year is equivalent to nine mice days (35), the extension of tPA therapeutic window to 4.5h post-injury in MACO mice would be considered a significant extension of tPA therapeutic window for human ischemic stroke. Thus, our results demonstrate the beneficial effects of IFNβ on the extension of tPA therapeutic window for ischemic stroke treatment.

Studies have shown that MG-mediated inflammatory response is an important pathological mechanism in ischemic stroke (36, 37). MG can be activated by various stimuli to secrete factors that exert proinflammatory or anti-inflammatory effects (38, 39). Previous studies show that inflammatory MG exert detrimental effects, whereas anti-inflammatory MG possess neuroprotective effects in the CNS (8, 9). Thus, it is beneficial to promote anti-inflammatory and inhibit inflammatory phenotypes of MG in ischemic stroke. Since cerebral ischemia induces MG activation and tPA was shown to further promote MG activation (2, 3), we assessed whether IFNβ modulated MG phenotypes to confer protection against delayed tPA-exacerbated brain injury in ischemic stroke. Indeed, the effect of IFNβ on the induction of anti-inflammatory myeloid cells has been documented previously. For instance, IFNβ has been shown to induce anti-inflammatory macrophages that express M2 genes, including Arg1 and Fizz1 (40). In addition, studies reported that IFNAR1 signaling is required for LPS-induced IL-10 upregulation in macrophages (41). Furthermore, we have previously demonstrated that IFNβ exerts differential effects on inflammatory and anti-inflammatory cytokine production, leading to the suppression of proinflammatory cytokines, IL-12 and IL-23, and the promotion of anti-inflammatory cytokine IL-10 in dendritic cells (42). Moreover, TLR3-induced upregulation of IL-10 was reported to be mediated by IFNβ in MG (43). Finally, the production of IFNβ by macrophages has been shown to promote the resolution of bacterial inflammation, and this effect is potentially mediated through IFNβ-induced IL-10 production in macrophages *in vivo* (44). In the present study, our findings revealed that IFNβ attenuated tPA-upregulated inflammatory molecules, CD86, IL-1α, and IL-1β, and promoted anti-inflammatory molecules, Arg1 and CD206, in MG during the acute phase of ischemic stroke. Notably, the effects of IFNβ on modulating MG phenotypes and ameliorating delayed tPA-exacerbated brain injury in ischemic stroke were abolished in mice with the conditional knockdown of IFNAR1 in MG. Finally, our *in vitro* studies showed that IFNβ suppressed TNFα+tPA-induced inflammatory molecules, IL-1α and IL-1β, and enhanced anti-inflammatory molecules, Arg1 and CD206, in primary MG, consistent with our *in vivo* findings. Altogether, our results provide the first evidence that IFNβ exerts modulatory effects on suppressing inflammatory and promoting anti-inflammatory MG, and that may subsequently lead to attenuated neuroinflammation and ameliorated brain injury in delayed tPA-treated ischemic stroke.

Previous studies showed that infiltrating monocytes/macrophages become MG-like phenotypes following phagocytosis, leading to the conversion of CD45^hi^ macrophages into CD45^int^ macrophages, and this conversion was observed in the ischemic brain of stroke animals at the later time points of day 3 and day 7 post-injury (27–29). In this study, we determined MG based on their intermediate expression of CD45 and positive expression of CD11b, and the expression of pro-inflammatory and anti-inflammatory markers in CD45^int^CD11b^+^ MG was assessed at day 1 and day 2 post-injury, respectively. We verified whether the population of CD45^int^CD11b^+^ cells contained infiltrating macrophages by measuring the expression of CD11a and Ly6C in CD45^int^CD11b^+^ cells, as these two markers are well defined surface markers for macrophages (28, 45, 46). We did not observe CD45^int^CD11b^+^ cells expressed CD11a or Ly6C in the ischemic brain of day 1 MCAO mice. However, a slight increase of Ly6C^+^ cells in the population of CD45^int^CD11b^+^ cells was observed in the ischemic brain of day 2 MCAO mice. Interestingly, a further increased frequency of Ly6C^+^CD45^int^CD11b^+^ cells was found in the ischemic brain of tPA-treated mice compared to that of vehicle- or tPA+IFNβ-treated MCAO mice. We speculated that increased Ly6C^+^CD45^int^CD11b^+^ cells in the ischemic brain of tPA-treated MCAO might be due to increased cell infiltrates converting into MG-like phenotypes following reperfusion. Notably, we observed that Arg1^+^ cells were largely associated with Ly6C^-^CD45^int^CD11b^+^ cells but not Ly6C^+^CD45^int^CD11b^+^ cells in the ischemic brain of vehicle-, tPA-, and tPA+IFNβ-treated MCAO mice. Taken altogether, our findings suggest that ischemic stroke promotes a low frequency of infiltrating Ly6C^+^CD45^hi^CD11b^+^ cells converting into Ly6C^+^CD45^int^CD11b^+^ MG-like cells in the ischemic brain at day 2 but not day 1 post-injury. Most importantly, we found that IFNβ-mediated attenuation of inflammatory phenotype (day 1) and augmentation of anti-inflammatory phenotype (day 2) of MG in tPA-treated MCAO mice were largely associated with Ly6C^-^CD45^int^CD11b^+^ resident MG but not Ly6C^+^CD45^int^CD11b^+^ MG-like infiltrating cells. Thus, our findings suggest that IFNβ mainly modulates resident MG phenotypes and that may then alleviate tPA-aggravated neuroinflammation in ischemic stroke.

The BBB possesses an essential role in maintaining the CNS homeostasis. The integrity of BBB is maintained by physical and biochemical characteristics, including tight junction protein complexes and transporters (47). In addition, its function is supported by astrocytes, pericytes, neurons, and MG that form the neurovascular unit (NVU). The cellular components of NVU can be activated upon sterile inflammation or disease induction that contributes to the BBB remodeling (48). This is particularly true for MG, because MG can be polarized into inflammatory or anti-inflammatory phenotype that exerts different impacts on BBB integrity. Studies have shown that inflammatory MG promote BBB dysfunction, whereas anti-inflammatory MG contribute to maintaining BBB integrity (49–51). Notably, our results showed that delayed tPA treatment promoted the inflammatory phenotype of MG, and that was correlated with aggravated BBB disruption in MCAO mice with delayed tPA treatment. In contrast, IFNβ suppressed tPA-enhanced inflammatory MG and promoted anti-inflammatory MG, and that was linked to lessened BBB disruption in delayed tPA-treated MCAO mice. Importantly, we found that the effect of IFNβ on inflammatory MG suppression and anti-inflammatory MG promotion was abrogated in delayed tPA-treated MCAO mice with MG-specific IFNAR1 deletion. Interestingly, we observed the protective effect of IFNβ on lessening delayed tPA-aggravated BBB disruption was partly but not fully abolished in these animals. Altogether, these results suggest that IFNβ-mediated modulation of MG phenotypes contributes to the alleviation of BBB disruption in ischemic stroke with delayed tPA treatment.

There are limitations with our current study. First, further studies would be required to identify the signaling pathways activated by IFNβ/IFNAR1 axis modulating MG polarization in delayed tPA-treated ischemic stroke. IFNAR1 signaling activates STAT1, STAT2, and STAT3. Studies have shown that STAT1 and STAT2 mediate the antiviral and inflammatory effects of IFNAR1 signaling (52–55). However, IFNAR1 signaling-induced STAT3 activation has been shown to inhibit STAT1-dependent gene activation, thereby downregulating IFNAR1 activation-induced inflammatory mediators (52). In addition, STAT3 activation has been shown to promote Arg1 and IL-10 upregulation (56). Thus, it is possible that IFNAR1 signaling induces STAT3 activation, leading to the suppression of tPA-enhanced inflammatory molecules, IL-1α, IL-1β and CD86, expression and the promotion of anti-inflammatory molecules, Arg1 and CD206, upregulation in MG in delayed tPA-treated stroke animals. Further studies of silencing STAT3 specifically in MG would provide insight into whether the activation of STAT3 *via* IFNAR1 signaling is required for IFNβ-mediated modulation of MG phenotypes in ischemic stroke with delayed tPA treatment. Second, we observed that the protective effect of IFNβ on lessening delayed tPA-aggravated BBB in ischemic stroke was partly reversed in MCAO mice with MG-specific IFNAR1 knockdown, suggesting that IFNβ-mediated modulation of MG phenotypes plays a role in lessening delayed tPA-aggravated BBB disruption in ischemic stroke. Since IFNAR1 is highly expressed in brain endothelial cells (57, 58), it is possible that IFNβ acts on brain endothelial cells to offer partial protection in MG-specific IFNAR1 knockdown MCAO mice subjected to delayed tPA treatment. Indeed, our previous study showed that IFNβ attenuated adhesion molecules, ICAM-1 and E-selectin, upregulation in TNFα-stimulated bEnd.3 cells *in vitro* and in the ischemic brain *in vivo* (13), suggesting IFNβ exerts a direct effect on the suppression of brain endothelial cell activation. Thus, further studies using conditional knockout mice in which IFNAR1 is specifically knockout in brain endothelial cells are warranted to elucidate the direct effect of IFNβ on modulating brain endothelial cells to lessen delayed tPA-aggravated BBB disruption in ischemic stroke. Finally, in this study we used the intraluminal suture MCAO model to evaluate the effect of IFNβ on the amelioration of delayed tPA-exacerbated brain injury in ischemic stroke. It is recognized that the suture MCAO model produces stable and consistent outcomes of ischemic brain injury. Thus, this model benefits our cellular and molecular mechanism studies to decipher the protective effect of IFNβ on ameliorating delayed tPA-exacerbated brain injury in ischemic stroke. Nevertheless, the suture MCAO model has limitations on assessing tPA thrombolytic effects *in vivo*. Thus, studies utilizing the thromboembolic MCAO model would be needed to further confirm the observed mechanism of IFNβ-exerted modulation of MG phenotypes in delayed tPA-treated MCAO mice.

In summary, we report that IFNβ extends tPA therapeutic window to 4.5h post-injury in ischemic stroke. Furthermore, we show that IFNβ exerts protective effects on attenuating delayed tPA-exacerbated brain injury, lessening delayed tPA-aggravated BBB disruption, and reducing delayed-tPA enhanced mortality in ischemic stroke. Mechanistically, we identify that IFNβ modulates MG phenotypes, leading to the suppression of inflammatory MG and the promotion of anti-inflammatory MG in delayed tPA-treated MCAO animals. Importantly, we find that MG-mediated modulation of MG phenotypes plays an important role in ameliorating delayed tPA-exacerbated ischemic brain injury, because the knockdown of IFNAR1 specifically in MG abrogates the effect of IFNβ on modulating MG polarization, abolishes the protective effect of IFNβ on ameliorating brain injury, and partly reverses the beneficial effect of IFNβ on lessening BBB disruption in delayed tPA-treated MCAO animals. In summary, our current study reveals a novel function of IFNβ on the modulation of MG polarization toward anti-inflammatory phenotype that may subsequently confer protection against delayed tPA-exacerbated brain injury in ischemic stroke. Thus, our findings open a new venue for the use of IFNβ in the combination of tPA to attenuate neuroinflammation and extend tPA therapeutic window in ischemic stroke.

## Data availability statement

The original contributions presented in the study are included in the article/**Supplementary Material**. Further inquiries can be directed to the corresponding author.

## Ethics statement

The animal study was reviewed and approved by Purdue Animal Care and Use Committee.

## Author contributions

P-CK performed the experiments, analyzed data, and wrote the manuscript. W-TW, BS, HP, PB, and BK performed the experiments. I-CIY reviewed and edited the manuscript. J-HJY. conceived the study, designed the experiments, and wrote the manuscript. All authors contributed to the article and approved the submitted version.
