# Supplementary material for: Interferon-β modulates microglial polarization to ameliorate delayed tPA-exacerbated brain injury in ischemic stroke
Source: Front Immunol. 2023 Mar 31;14:1148069. doi: 10.3389/fimmu.2023.1148069 (PMC10104603; doi:10.3389/fimmu.2023.1148069)
Supplement: Supplementary file 1 [file DataSheet_1.pdf]

Supplementary Figure 1

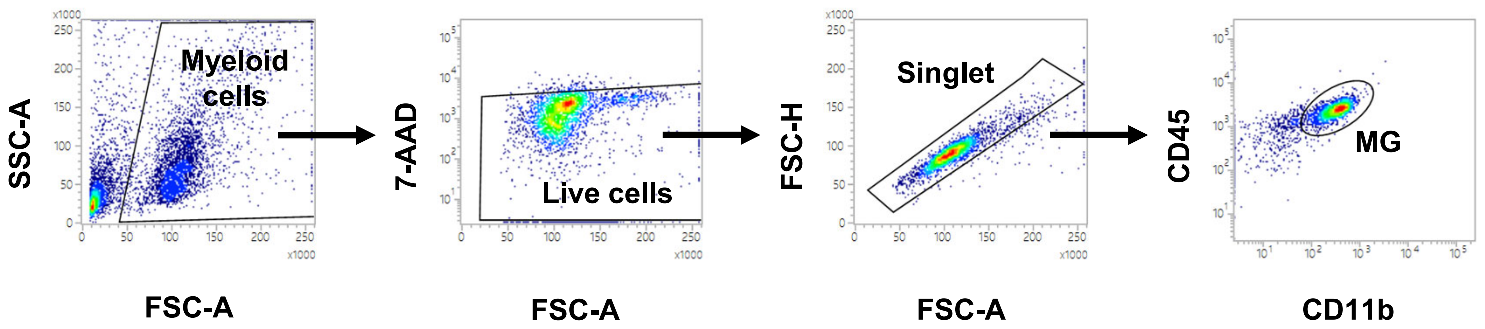

Supplementary Figure 1 Gating strategy of flow cytometry analysis. Mononuclear cells isolated from the brains of sham and MCAO mice were subjected to surface staining of CD11b and CD45 antibodies in the presence of 7-AAD. 7-AAD negative live cells were then gated followed by singlet gating. MG were gated based on their intermediate expression of CD45 and positive expression of CD11b.

Supplementary Figure 2

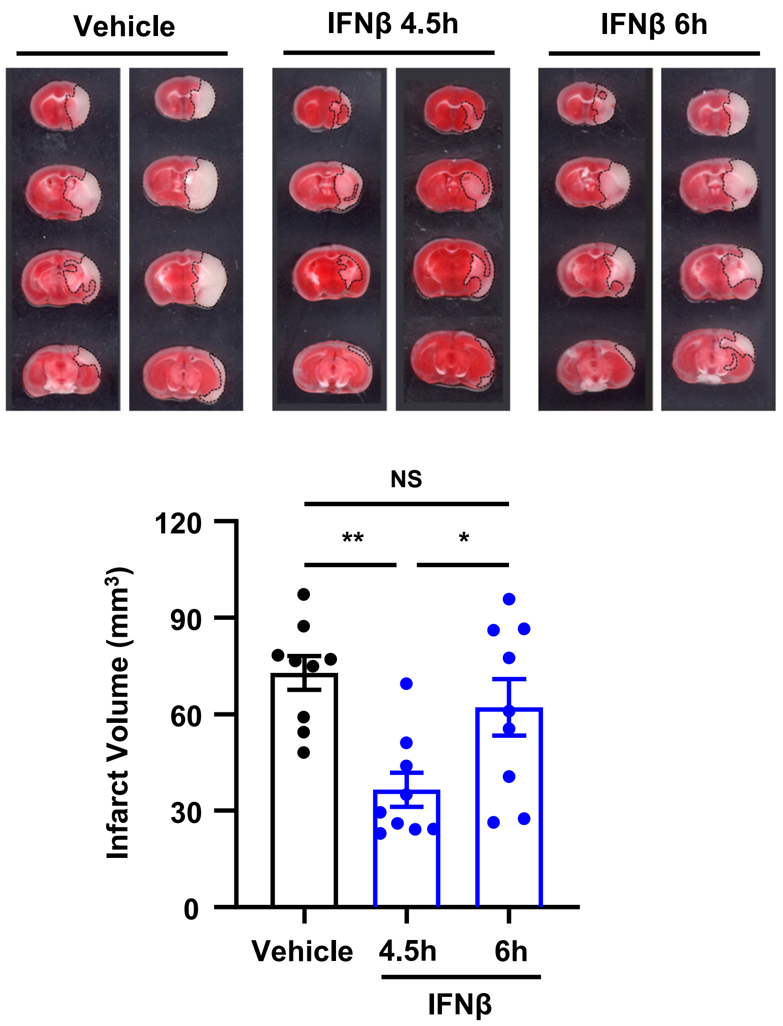

Supplementary Figure 2 IFNβ administered at 4.5h but not 6h post-reperfusion confers protection against ischemic stroke. C57BL/6 mice were subjected to MCAO followed by IFNβ administration at 4.5h or 6h post-reperfusion (n=9/group). At day 2 post-injury, the ischemic brains were harvested and subjected to TTC staining. Two representative TTC-stained brain samples of each group are shown, and the infarct volumes were measured. *$*p<0.05$; $**p<0.01$*; NS: no significant difference by one-way ANOVA.

Supplementary Figure 3

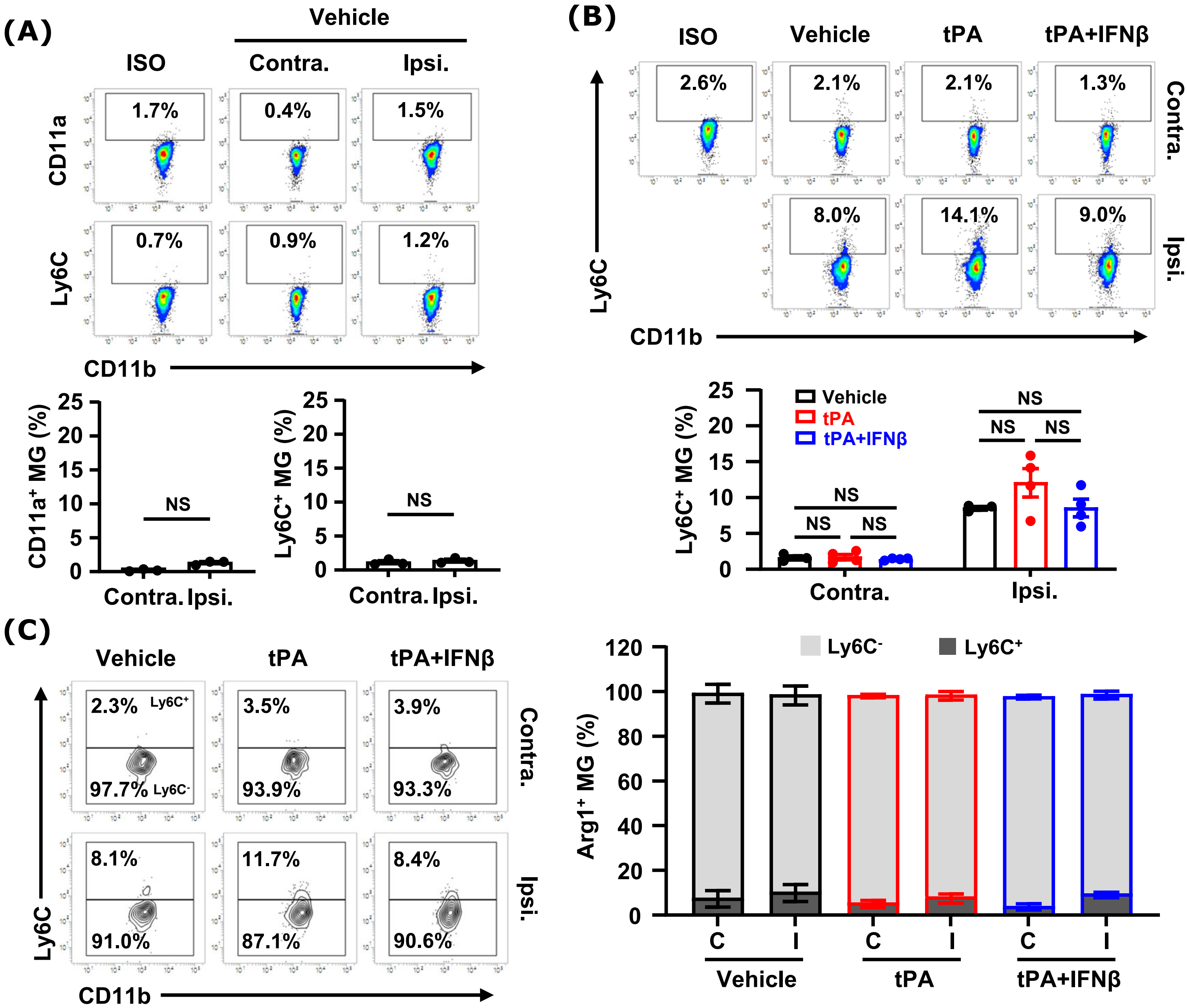

Supplementary Figure 3 Arg1⁺ cells are associated with Ly6C⁻CD45ⁱⁿᵗCD11b⁺ MG in the ischemic brain. (A) Male C57BL/6 mice were subjected to 40min MCAO. At day 1 post-injury, the contralateral (Contra.) and ipsilateral (Ipsi.) hemispheres of MCAO mice (n=3) were harvested followed by mononuclear cell isolation. The isolated cells were stained with CD45 and CD11b antibodies in the presence of CD11a and Ly6C antibodies followed by flow cytometry analysis. The frequency of $CD11a^+$ and $Ly6C^+$ cells in $CD45^{int}CD11b^+$ cells was determined. (B and C) Male C57BL/6 mice were subjected to 40min MCAO followed by the administration of vehicle (n=3), tPA (n=4), or tPA+IFNβ (n=4) at 4.5h post-reperfusion. At day 2 post-injury, the contralateral (Contra.; C) and ipsilateral (Ipsi.; I) hemispheres of vehicle-, tPA-, and tPA+IFNβ-treated MCAO mice were harvested followed by mononuclear cell isolation. The isolated mononuclear cells were stained with CD45 and CD11b antibodies in the presence of Ly6C antibody. Following fixation and permeabilization, cells were then stained with Arg1 antibody followed by flow cytometry analysis. (B) The frequency of $Ly6C^+$ cells in $CD45^{int}CD11b^+$ cells was determined. (C) $Arg1^+CD45^{int}CD11b^+$ cells were gated, and the frequency of $Ly6C^+$ and $Ly6C^-$ cells in $Arg1^+CD45^{int}CD11b^+$ cells was determined. NS: no significant difference by Mann-Whitney *U* test (A) or one-way ANOVA (B).

Supplementary Figure 4

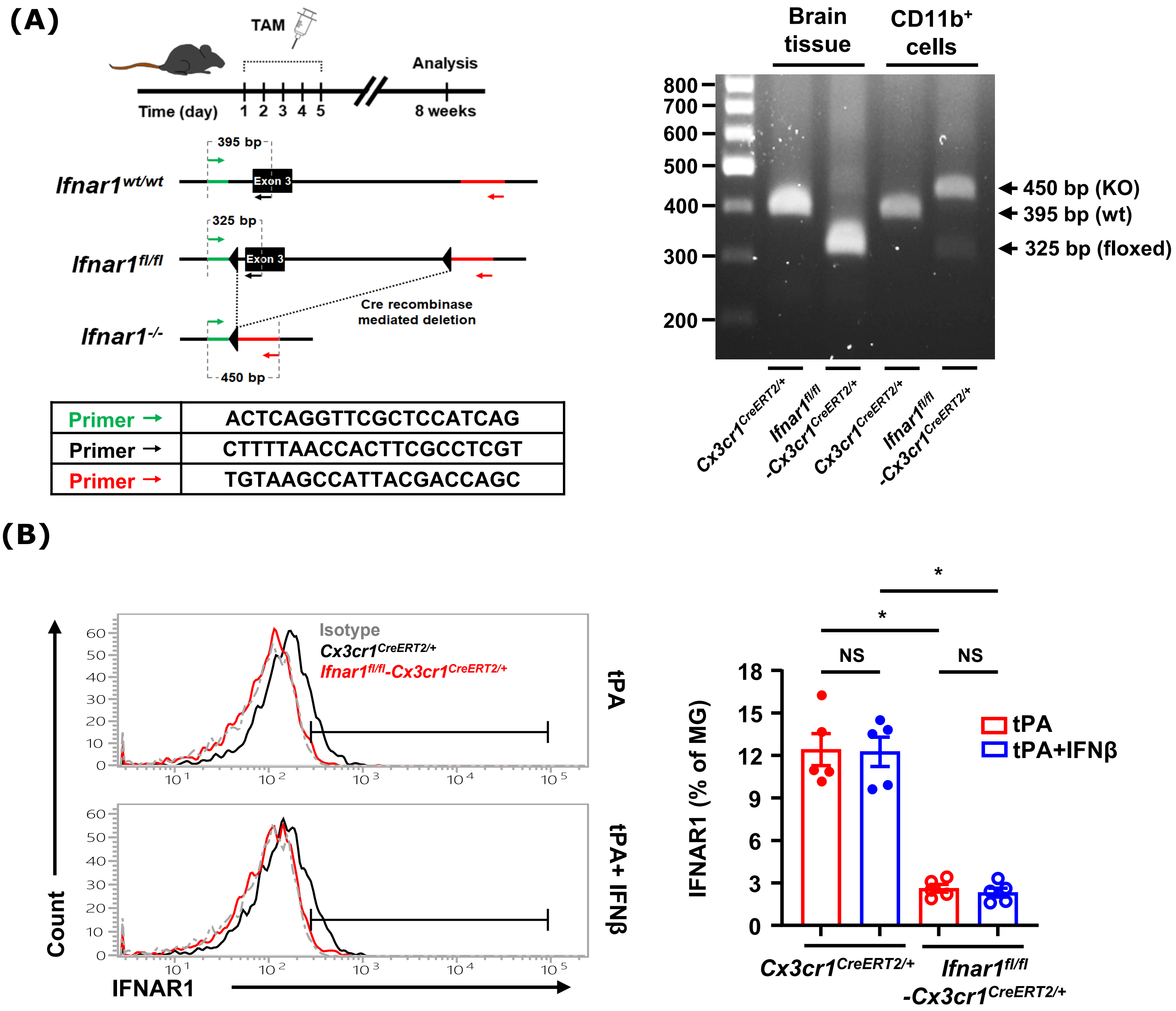

Primer →	ACTCAGGTTCGCTCCATCAG
Primer →	CTTTTAACCACTTCGCCTCGT
Primer →	TGTAAGCCATTACGACCAGC

Supplementary figure 4 MG-specific knockdown of IFNAR1 in TAM-treated *Ifnar1$^{fl/fl}$-Cx3cr1$^{CreERT2/+}$* mice. (A) The genomic DNA extracted from whole brain tissue and CD11b^{+} cells isolated form the brains of *Cx3cr1$^{CreERT2/+}$* and *Ifnar1$^{fl/fl}$-Cx3cr1$^{CreERT2/+}$* mice were subjected to multiplex PCR genotyping at 8 weeks after TAM treatment. The PCR primers used are listed in the table. (B) *Cx3cr1$^{CreERT2/+}$* and *Ifnar1$^{fl/fl}$-Cx3cr1$^{CreERT2/+}$* mice were subjected to MCAO followed by the treatment of tPA in the presence or absence of IFNβ at 4.5h post-reperfusion (n=5/group). At day 2 post-injury, the ischemic brains were harvested followed by mononuclear cell isolation, and the isolated cells were subjected to staining with CD45 and CD11b antibodies in the presence of IFNAR1 antibody. The expression of IFNAR1 in CD45^{int}CD11b^{+} MG was then determined. *$p<0.05$; NS: no significant difference by one-way ANOVA.
